# California

**Published:** 1888-08

**Authors:** 


CALIFORNIA.—The annual death rate in California is put down at 16.8. Consumption is placed at 18 82 of total mortality; due largely to the influx of invalids from the Eastern States. In San Francisco the annual death rate is 20. In Los Angeles the death rate is only 9.6. In San Diego the death rate 18.6. In Sacramento, 11,6.

